# Investigation of Structural, Morphological and Magnetic Properties of MFe_2_O_4_ (M = Co, Ni, Zn, Cu, Mn) Obtained by Thermal Decomposition

**DOI:** 10.3390/ijms23158483

**Published:** 2022-07-30

**Authors:** Thomas Dippong, Erika Andrea Levei, Oana Cadar

**Affiliations:** 1 Faculty of Science, Technical University of Cluj-Napoca, 76 Victoriei Street, 430122 Baia Mare, Romania; dippong.thomas@yahoo.ro; 2 INCDO-INOE 2000, Research Institute for Analytical Instrumentation, 67 Donath Street, 400293 Cluj-Napoca, Romania; erika.levei@icia.ro

**Keywords:** ferrite, thermal decomposition, heating temperature, crystalline phase, specific surface, magnetic behavior

## Abstract

The structural, morphological and magnetic properties of MFe_2_O_4_ (M = Co, Ni, Zn, Cu, Mn) type ferrites produced by thermal decomposition at 700 and 1000 °C were studied. The thermal analysis revealed that the ferrites are formed at up to 350 °C. After heat treatment at 1000 °C, single-phase ferrite nanoparticles were attained, while after heat treatment at 700 °C, the CoFe_2_O_4_ was accompanied by Co_3_O_4_ and the MnFe_2_O_4_ by α-Fe_2_O_3_. The particle size of the spherical shape in the nanoscale region was confirmed by transmission electron microscopy. The specific surface area below 0.5 m^2^/g suggested a non–porous structure with particle agglomeration that limits nitrogen absorption. By heat treatment at 1000 °C, superparamagnetic CoFe_2_O_4_ nanoparticles and paramagnetic NiFe_2_O_4_, MnFe_2_O_4_, CuFe_2_O_4_ and ZnFe_2_O_4_ nanoparticles were obtained.

## 1. Introduction

Spinel ferrites of MFe_2_O_4_ (M = Co, Ni, Zn, Cu, Mn) type have a cubic, closely packed arrangement of oxygen atoms with M^2+^ and Fe^3+^ ions occupying the tetrahedral (A) and octahedral (B) sites [1]. The spinel structure determines excellent magnetic and electrical properties, high chemical stability and low production costs [1,2,3,4,5,6,7,8,9,10]. These interesting properties enable the use of nanostructured materials in a wide range of novel applications in the field of science and technology [9,10,11,12,13].

Cobalt ferrite (CoFe_2_O_4_), nickel ferrite (NiFe_2_O_4_) and copper ferrite (CuFe_2_O_4_) have inverse spinel structures with M^2+^ (M^2+^ = Co^2+^, Ni^2+^ or Cu^2+^) ions occupying the octahedral (B) sites and Fe^3+^ ions equally distributed between the tetrahedral (A) and octahedral (B) sites [5,14]. Zinc ferrite (ZnFe_2_O_4_) has a normal spinel ferrite with Zn^2+^ ions in tetrahedral (A) and Fe^3+^ ions in octahedral (B) sites, while manganese ferrite (MnFe_2_O_4_) has a partially inverse spinel structure, in which only 20% of divalent Mn^2+^ ions are located at octahedral (B) sites and the remainder of them are positioned at tetrahedral (A) sites [6]. 

CoFe_2_O_4_ is a ferromagnetic material with unique characteristics, such as large coercivity, magnetocrystalline anisotropy, Curie temperature and electrical resistance, remarkable thermal stability, moderate saturation magnetization, good chemical and mechanical stability, low eddy current loss and production cost [2,3,11,15]. These properties make it a promising candidate for various kind of applications such as drug delivery, magnetic resonance imaging, magnetic storage devices, catalysts and adsorption of toxic metals [15,16,17,18,19]. CoFe_2_O_4_ with unique architectures, including nanoparticles, hollow nanospheres, mesoporous nanospheres, nanorods and three-dimensional ordered macroporous structures, have been produced in the last years [3]. 

NiFe_2_O_4_ may display paramagnetic, superparamagnetic or ferrimagnetic behavior depending on the particle size and shape [20]. Due to its high magnetocrystalline anisotropy, high-saturation magnetization and unique magnetic structure combined with high Curie temperature, low coercivity, low eddy current loss, low price and high electrochemical stability [12,20,21,22] it is one of the most suitable candidates for applications in biosensors, corrosion protection, drug delivery, ceramics, medical diagnostics, microwave absorbers, transformer cores, magnetic liquids, magnetic refrigeration and high-density magnetic recording media, water-oxidation processes, dye removal by magnetic separation, etc. [5,21,22,23].

CuFe_2_O_4_ is a soft material with low coercivity, low-saturation magnetization, high electrical resistance, low eddy current losses, great resistance to corrosion, thermal stability, excellent catalytic properties and environmental benignity, and it is not readily demagnetized [12,14,24,25]. CuFe_2_O_4_ shows ferromagnetic behavior with a single-domain state and is widely used in magnetic storage, catalysis, photocatalysis, pollutant removal from wastewater, color imaging, magnetic refrigeration, magnetic drug delivery and high-density information storage [8,12,24,25,26]. 

ZnFe_2_O_4_ possess exceptional structural, optical, magnetic, electrical and dielectric properties at nanoscale, besides low toxicity, chemical and thermal stability [5,7,9,11,15]. ZnFe_2_O_4_ is antiferromagnetic at temperatures below the Neel temperature, but when the size of ZnFe_2_O_4_ approaches the nanometer range, it transforms into a diamagnetic, superparamagnetic or ferromagnetic substance [12,18]. Consequently, it has a wide potential to be used in microwave absorption, energy storage, drug delivery, magnetic resonance imaging, gas sensors, absorbent material for hot-gas desulphurization, high-performance electrode materials, photocatalysts and pigments [5,9,11,12,27]. Additionally, ZnFe_2_O_4_ is a promising semiconductor that can sensitize and activate under visible light other photocatalysts due to its small band gap [28].

MnFe_2_O_4_ have controllable grain size, high magnetization value, superparamagnetic nature, ability to be monitored by an external magnetic field, an easy synthesis process, surface manipulation ability, greater biocompatibility, thermal stability, non–toxicity, noncorrosion and environmentally friendly ability. Its properties have attracted potential consideration in biomedicine, in ceramic and paint industry as black pigment, and in high-frequency magnetostrictive and electromagnetic applications [6,29,30].

Synthesis methods with low toxicity that are also economical in terms of energy consumption, allowing for the production of fine, nanosized, highly pure, single-phase nanocrystalline ferrites have received considerable interest [31]. Spinel ferrites are commonly synthesized using the ceramic technique, which infers high temperatures and produces particles with small specific surface. In order to achieve ferrites with large specific surface and high degree of homogeneity, different synthesis methods, namely coprecipitation, polymeric gel, hydrothermal, microemulsion, heterogeneous precipitation, sonochemistry, combustion, sol–gel methods, etc. were used [31]. Despite the resulting fine-grained microstructure, the chemical methods have some disadvantages such as necessity of complex apparatus, long reaction time and post–synthesis thermal treatment to complete the formation and crystallization of final products, poor crystallinity and broad particle size distribution, which may negatively influence the related properties (shape, surface area and porosity) [32]. The sol–gel method has been used to prepare fine, homogenous, highly dense and single-phase ferrite nanoparticles. Compared to other conventional methods, the sol–gel method provides a good stoichiometric control and produces ferrites at relatively low temperatures. Furthermore, it allows for the embedding of ferrites into silica (SiO_2_) matrix to prevent particle growth and particle agglomeration and to improve the magnetic properties [16,33]. However, despite its noticeable advantages, its main disadvantage consists of limited efficiency and long processing time [34]. The thermal decomposition method is a very efficient synthesis strategy, based on the heating of metallic precursors at different temperatures. Additionally, this method is simple and environmentally friendly, has a relatively low cost, requires a low reaction temperature and provides small particle size, narrow size distribution and no toxic by-products [35].

This paper focuses on the structural and morphological characteristics as well as the magnetic properties of nanosized CoFe_2_O_4_, NiFe_2_O_4_, ZnFe_2_O_4_, CuFe_2_O_4_ and MnFe_2_O_4_, obtained by thermal decomposition at 700 and 1000 °C. To the best of our knowledge, this is the first work that investigates the structural, morphological and magnetic properties of nanoferrites obtained by thermal decomposition of nitrates and compares them with those of correspondent nanoferrites embedded in SiO_2_ matrix obtained by sol–gel method. The reaction progress was monitored by thermal (TG/DTA) analysis, while the nanoferrite composition was investigated by inductively coupled plasma optical emission spectrometry (ICP-OES). The crystalline phases and crystallite size were investigated by X-ray diffraction (XRD), while the particle properties such as shape, size and agglomeration were studied by transmission electron microscopy (TEM). The influence of crystallite size and divalent ions on the magnetic properties and the variation of saturation magnetization, remanent magnetization, coercivity and anisotropy of nanoferrites were also studied. 

## 2. Results

**Figure 1:**
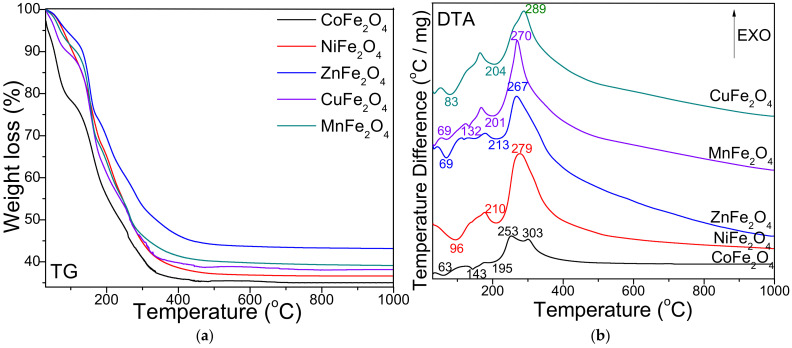
Thermogravimetric (TG) (**a**) and differential thermal analysis (DTA) (**b**) diagrams for MFe_2_O_4_ (M = Co, Ni, Zn, Cu, Mn).

Figure 1 presents the thermal decomposition diagrams (thermogravimetric—TG and differential thermal—DTA) of MFe_2_O_4_ systems. On the DTA diagram, the formation of CoFe_2_O_4_ is indicated by three endothermic effects at 63, 143 and 195 °C and two exothermic effects at 253 and 303 °C, respectively. The total mass loss is 65%. NiFe_2_O_4_, ZnFe_2_O_4_ and CuFe_2_O_4_ show two endothermic effects at 96 and 210 °C, 69 and 213 °C, 83 and 204 °C and an exothermic effect at 279, 267 and 289 °C, respectively. The total mass loss is 63% for NiFe_2_O_4_, 57% for ZnFe_2_O_4_ and 62% for CuFe_2_O_4_, respectively. The formation of MnFe_2_O_4_ is showed by three endothermic effects at 69, 132 and 201 °C, and an exothermic effect at 270 °C. The total mass loss shown on the TG diagram is 60%. 

**Figure 2:**
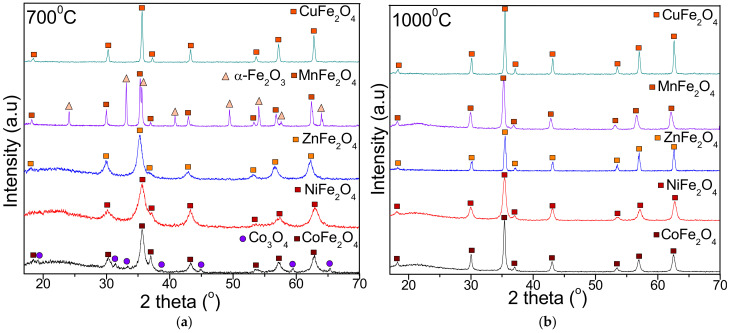
X-ray diffraction pattern of MFe_2_O_4_ (M = Co, Ni, Zn, Mn, Cu) heat treated at 700 °C (**a**) and 1000 °C (**b**).

The crystalline phases after heat treatment at 700 and 1000 °C are presented in Figure 2. The XRD pattern of CoFe_2_O_4_ exhibits a single-phase cubic spinel CoFe_2_O_4_ (JCPDS card no. 22-1086, [36]), belonging to *Fd3m* space group at 1000 °C, while at 700 °C, the presence of Co_3_O_4_ (JCPDS card no. 80-1451 [36]) is also remarked. In case of NiFe_2_O_4_, ZnFe_2_O_4_ and CuFe_2_O_4_ at both temperatures, single phase crystalline NiFe_2_O_4_ (JCPDS card no. 89-4927 [36]), ZnFe_2_O_4_ (JCPDS card no. 16-6205 [36]) and CuFe_2_O_4_ (JCPDS card no. 25-0283 [36]) are remarked. The presence of ZnO or CuO identified in case of ferrites embedded in SiO_2_ matrix was not observed [33]. Single-phase crystalline MnFe_2_O_4_ (JCPDS card no. 74-2403 [36]) is obtained at 1000 °C, while at 700 °C, the MnFe_2_O_4_ is accompanied by α-Fe_2_O_3_ (JCPDS card no. 87-1164 [36]).

The average crystallite size was estimated using the most intense diffraction (311) peak from the Debye–Scherrer formula [7,23,25] (Table 1). The crystallite size increases with the heating temperature, with the largest crystallite size being observed for CuFe_2_O_4_ at 1000 °C (81 nm), while the smallest crystallite size was observed for ZnFe_2_O_4_ at 700 °C (13 nm). The lattice parameter (a) also increases with the annealing temperature, with the highest value being observed for NiFe_2_O_4_ at 1000 °C (8.365 Å), while the lowest value observed was for CuFe_2_O_4_ at 700 °C (8.207 Å). The M/Fe molar ratio calculated based on Co, Mn, Zn, Ni and Fe concentrations measured by ICP-OES confirms the theoretical elemental composition of the obtained nanoferrites (Table 1). In all cases, the best fit of experimental and theoretical data is remarked for samples annealed at 1000 °C. In case of CoFe_2_O_4_ and MnFe_2_O_4_ annealed at 700 °C, the M/Fe molar ratio could not be calculated due to the presence of Co_3_O_4_ and α-Fe_2_O_3_ as secondary phases.

Due to the low amount of adsorbed/desorbed nitrogen, the determination of porosity and specific surface area (SSA) for samples heat treated at 700 and 1000 °C was not possible. The SSA below the method detection limit (0.5 m^2^/g), suggests that all ferrites have a non–porous structure, probably as a consequence of particle agglomeration that limits the absorption of nitrogen.

**Figure 3:**
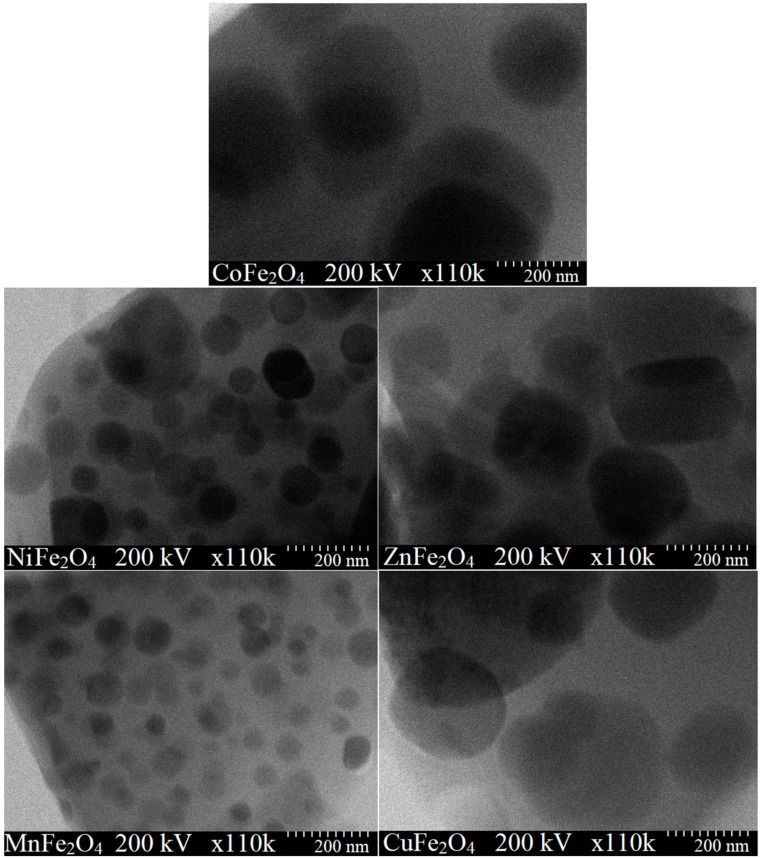
TEM images for MFe_2_O_4_ (M = Co, Zn, Ni, Cu, Mn) heat treated at 1000 °C.

According to TEM images, the nanoparticles have spherical shape. The particle sizes estimated by XRD and TEM are comparable, the low differences appearing probably due to some large-size nanoparticles. CuFe_2_O_4_ displays the largest particle size (85 nm), while MnFe_2_O_4_ has the smallest particle size (32 nm) (Table 1 and Figure 3).

**Figure 4:**
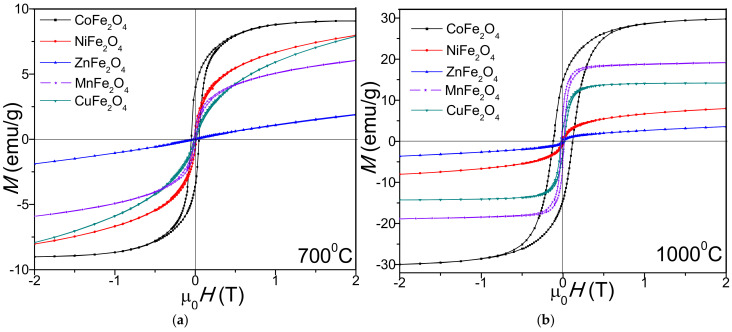
Magnetic hysteresis loops of MFe_2_O_4_ (M = Co, Zn, Ni, Cu, Mn) heat treated at 700 °C (**a**) and 1000 °C (**b**).

The hysteresis loops (Figure 4) have an S-shape at low magnetic fields and are linear at higher fields, indicating the presence of small-sized magnetic particles with superparamagnetic behavior [37]. The spin rotation energy for particles smaller than the critical diameter is lower than the thermal energy. Thus, in the absence of an applied magnetic field, the random orientation of the magnetic moments results in zero average global magnetic moment [36].

The saturation magnetization (M_S_) and remanent magnetization (M_R_) increase with the increase of heating temperature, with the highest values being measured for CoFe_2_O_4_ (29.7 emu/g) and the lowest for ZnFe_2_O_4_ (2.45 emu/g), as shown in Table 2. The coercivity (H_C_) increases (from 49.5 Oe to 131 Oe in case of CoFe_2_O_4_) with the increase of heating temperature, indicating that the magnetic moment arrangement is highly disordered at high heating temperatures [27]. 

In all cases, the squareness ratio (S, 0.075 for ZnFe_2_O_4_ at 700 °C—0.727 for NiFe_2_O_4_ at 1000 °C), the anisotropy constant (K, 0.002 × 10^3^ erg/cm^3^ for ZnFe_2_O_4_ at 700 °C—2.44 × 10^3^ erg/cm^3^ for CoFe_2_O_4_ at 1000 °C) also increase with the increase of heating temperature. Compared to the same ferrites embedded in SiO_2_ matrix, the K value is much lower [33].

## 3. Discussion

The thermal behavior of Fe^III^(NO_3_)_3_–M^II^(NO_3_)_2_–1,3-propanediol solutions was studied by DTA. The endothermic effect at 70–100 °C is attributed to the loss of moisture and of crystallization water from the metallic nitrates used in the synthesis. The endothermic effect at 132–213 °C on the DTA diagram is attributed to formation of metal-malonate precursor. The formation of metal-malonate precursor for CoFe_2_O_4_ and ZnFe_2_O_4_ takes place in two stages as indicated by the two endothermic effects. The first endothermic effect was assigned to the divalent metal-malonate formation (143 °C for Co-malonate and 132 °C for Mn-malonate), while the second endothermic effect (around 201 °C) was assigned to the formation of Fe-malonate. In case of the other synthesis, the divalent metal (Ni, Zn, Cu) malonates and the trivalent Fe-malonate formation takes place in a single stage (204–213 °C). The formation of ferrites by decomposition of malonate precursors is indicated on the DTA diagram by a single exothermic effect at 250–350 °C, except for CoFe_2_O_4_, where two exothermic effects appear at 253 and 303 °C. The two-stage formation of CoFe_2_O_4_ in the metal nitrates–diol mixture could be explained by the fact that the aqua cation [Fe(H_2_O)_6_]^3+^ is a stronger acid than the aqua cation [Co(H_2_O)_6_]^2+^ [17,36]. The highest mass loss shown on the TG diagram is attributed to CoFe_2_O_4_ (65%), while the lowest mass loss is attributed to ZnFe_2_O_4_ (57%), probably due to the fact that ZnFe_2_O_4_ is quantitatively obtained at lower temperatures compared to other ferrites accompanied by other crystalline or amorphous secondary phases.

The increase of heating temperature from 700 to 1000 °C did not affect the crystal structure of the studied ferrites but improved the phase purity [10]. Moreover, by increasing the heating temperature, the diffraction peaks become sharper and narrower, indicating the formation of larger particles due to grain growth [25,38]. After heat treatment at 1000 °C, an important agglomeration of the particles takes place without consequent recrystallization, supporting the formation of single crystals rather than polycrystals [1,2,6]. Oppositely, at 700 °C, the surface dipole–dipole interactions, high surface energy and tension, as well as the change of cation distribution within the nanocrystallite, induces lattice shrinking, which further inhibits grain growth [1,2,3,4,5,6]. Generally, the size of the crystallite is higher than the size of the corresponding ferrites embedded in SiO_2_ matrix, produced by sol–gel method [15,16,17,18,19]. These findings indicate that the heating temperature and the synthesis route plays a key role in determining the crystallite size [8]. Crystallite size has a significant effect on the magnetic and optical properties of the material, especially when the grain size is approaching the crystallite size [4,5,6,38].

The different particle size of the produced ferrites may be attributed to different kinetics of metal oxides formation, different particle growth rate or presence of structural disorder and strain in the lattice caused by different ionic radii [39] The different particle arrangement is attributed to the formation of well-delimited particles that generate solid boundaries. Moreover, interfacial surface tensions appear most likely due to the agglomeration tendency of small particles, weak surface interaction due to Van der Waals forces and magnetic interactions [39].

The magnetic properties of nanoferrites are strongly influenced by the cation distribution between the tetrahedral (A) and octahedral (B) sites, as well as by the interactions between the magnetic ions [37,40]. Different size and morphology of the nanoparticles at different heating temperatures results in different surface spin disorder, pinned magnetic moment, different surface spin canting and consequently different cation inversion in the spinel structure and magnetic features [8]. The lower M_S_ values of ferrites heat treated at 700 °C compared to those at 1000 °C result from the lower crystallinity at 700 °C, presence of vacancies, interatomic spacing, low coordination number and surface spin disorder [37].

At 1000 °C, the CoFe_2_O_4_ has superparamagnetic behavior, while the other ferrites display paramagnetic behavior. The superparamagnetic behavior is attributed to the high disorder of the magnetic moment orientation with the increase in the surface-area-to-volume ratio [41]. For MnFe_2_O_4_, the sharp increase in M_S_ with the increase of heating temperature could be explained by the formation of trace paramagnetic α-Fe_2_O_3_ [27,30]. The ZnFe_2_O_4_ heat treated at 700 °C is paramagnetic, the low M_S_ values being attributed to the lattice defects, core–shell interactions, spin canting, disordered cation distribution, A–B super exchange interaction and random spin orientation on the surface of nanoparticles [30]. The M_S_ values reported for ZnFe_2_O_4_ differ from study to study, indicating that M_S_ strongly depends on the synthesis route and heating temperature [27,41]. The changes of CuFe_2_O_4_ magnetic properties following the reduction in bulk grain size of CuFe_2_O_4_ nanoparticles by milling was reported by Soufi et al. [12]. The M_S_ value of NiFe_2_O_4_ is lower than that of MnFe_2_O_4_ and CoFe_2_O_4_, probably due to the increase in surface effects with the decrease in particle size [20]. The influences of surface effect on the magnetic properties may be explained by the different exchange interactions and presence of magnetic defects on the nanoparticles surface [20]. Priyadharsini et al. also reported the M_S_, M_R_ and H_C_ values increasing with the heating temperature [25].

Generally, the H_C_ of the spinel ferrite nanoparticles is governed by the magnetocrystalline anisotropy, strain, interparticle interaction, grain size and morphology [42]. The H_C_ also increase with the increase in the surface potential barrier caused by crystalline lattice defects such as the deviation of atoms from the normal positions in the surface layers [43]. The influence of the particle sizes, internal strain, magnetic domain structure, shape and magnetocrystalline anisotropy of the nanoparticles on the H_C_ value is not fully explained [43]. The low H_C_ of all ferrites for both heating temperatures indicate an enhanced coalescence of the crystallites that further results in strong magnetic coupling and high magnetization [43]. At both temperatures, the H_C_ of CoFe_2_O_4_ nanoparticles prepared by thermal decomposition increases with the particle-size increase, suggesting the presence of a single magnetic domain [8]. The transition from superparamagnetic to ferromagnetic behavior of CoFe_2_O_4_ was noticed after heat treatment at 1000 °C [25]. The different H_C_ of NiFe_2_O_4_ is attributed both to the crystallite size and the presence of shape anisotropy [30].

The increasing squareness ratio (S) at high heating temperatures could be the consequence of the reorientation of grains along the easy axis of magnetization when the field is switched off [25]. The main factors that influence the magnetic anisotropy are the crystallographic directions, surface defects and irregularities [44,45]. The high H_C_ and K of CoFe_2_O_4_ are the consequence of Co^2+^ ions in octahedral (B) sites, which induce frozen orbital angular momentum and strong spin-orbital coupling [8,46]. 

## 4. Materials and Methods

Fe(NO_3_)_3_∙9H_2_O, Co(NO_3_)_2_∙6H_2_O, Ni(NO_3_)_2_∙6H_2_O, Zn(NO_3_)_2_·6H_2_O, Cu(NO_3_)_2_∙3H_2_O, Mn(NO_3_)_2_∙3H_2_O and 1,3-propanediol of purity higher than 98% were purchased from Merck (Darmstadt, Germany) and used as received. 

MFe_2_O_4_ (M = Co, Zn, Ni, Cu, Mn) were synthesized by mixing the metal nitrates in 1M/2Fe molar ratio with 1,3-propanediol in equimolecular ratio of NO_3_^−^/1,3-propanediol. The resulted solutions were heat treated at 700, and 1000 °C (5 h) in air using a LT9 muffle furnace (Nabertherm, Lilienthal, Germany).

The ferrite formation was investigated by thermogravimetry (TG) and differential thermal analysis (DTA) using a Q600 SDT (TA Instruments, New Castle, DE, USA) analyzer, in air, up to 1000 °C, at 10 °C·min^−1^ using alumina standards. The crystalline phases were investigated by X-ray diffraction using a D8 Advance (Bruker, Karlsruhe, Germany), at ambient temperature, with CuKα radiation (λ = 1.54060 Å) and LynxEye detector, operating at 40 kV and 40 mA. The Co/Fe (CoFe_2_O_4_), Ni/Fe (NiFe_2_O_4_), Zn/Fe (ZnFe_2_O_4_), Cu/Fe (CuFe_2_O_4_) and Mn/Fe (MnFe_2_O_4_) molar ratios were confirmed using an Optima 5300 DV (Perkin Elmer, Norwalk, CT, USA) inductively coupled plasma optical emission spectrometer (ICP-OES), spectrometer, after microwave digestion (Xpert microwave system, Berghof, Eningen, Germany) with aqua regia. Specific surface area (SSA) was calculated using the BET model from N_2_ adsorption–desorption isotherms recorded at 196 °C on samples degassed for 4 h at 150 °C and 2 Pa pressure using a Sorptomatic 1990 (Thermo Fisher Scientific, Waltham, MA, USA) instrument. The shape and clustering of nanoparticles were studied on samples deposited and dried on carbon-coated copper grids using a transmission electron microscope (TEM, HD-2700, Hitachi, Tokyo, Japan). The magnetic measurements were performed using a 7400 vibrating-sample magnetometer (VSM, LakeShore Cryotronics, Westerville, OH, USA). The hysteresis loops were recorded at room temperature in magnetic fields between −2 to 2 Tesla.

## 5. Conclusions

The structural, morphological and magnetic characteristics of nanosized CoFe_2_O_4_, NiFe_2_O_4_, ZnFe_2_O_4_, CuFe_2_O_4_ and MnFe_2_O_4_ obtained by thermal decomposition were investigated. The formation of ferrites appeared as a single exothermic effect at 250–350 °C, excepting CoFe_2_O_4_ with two exothermic effects. The highest mass loss was attributed to CoFe_2_O_4_ (65%), while the lowest mass loss was assigned to ZnFe_2_O_4_ (55%). Unlike similar ferrites embedded in SiO_2_ matrix, at both temperatures, single crystalline phases were remarked, excepting the presence of Co_3_O_4_ (in case of CoFe_2_O_4_) and α-Fe_2_O_3_ (in case of MnFe_2_O_4_) at 700 °C. The SSA values lower than 0.5 m^2^/g indicated a non–porous structure due to the particle agglomeration. CuFe_2_O_4_ showed the largest particle size (85 nm), while MnFe_2_O_4_ had the smallest particle size (32 nm). The crystalline CoFe_2_O_4_ heat treated at 1000 °C displayed the highest Ms, H_C_ and K values, presenting superparamagnetic behavior, while the other ferrites exhibited paramagnetic behavior.

## Figures and Tables

**Table 1:** Average particle size (D_PS_), average crystallite size (D_CS_), lattice parameter (a) and M/Fe molar ratio for MFe_2_O_4_ (M = Co, Ni, Zn, Mn, Cu) heat treated at 700 and 1000 °C.

	Temperature (°C)	CoFe_2_O_4_	NiFe_2_O_4_	ZnFe_2_O_4_	MnFe_2_O_4_	CuFe_2_O_4_
D_PS_ (nm)	1000	78	52	68	32	85
D_CS_ (nm)	700 1000	23 69	18 49	13 57	14 29	27 81
a (Å)	700 1000	8.275 8.334	8.258 8.365	8.278 8.342	8.269 8.318	8.207 8.302
M/Fe molar ratio	700 1000	- 0.99/2.00	0.98/2.04 0.99/2.01	0.97/2.01 0.99/2.00	- 0.98/1.99	0.98/2.03 1.00/2.01

**Table 2:** Saturation magnetization (M_S_), remanent magnetization (M_R_), coercivity (H_C_), squareness (S) and magnetic anisotropy constant (K) of MFe_2_O_4_ (M = Co, Zn, Ni, Cu, Mn) heat treated at 700 and 1000 °C.

	Temperature (°C)	CoFe_2_O_4_	NiFe_2_O_4_	ZnFe_2_O_4_	CuFe_2_O_4_	MnFe_2_O_4_
M_S_ (emu/g)	700 1000	9.20 29.7	8.08 11.2	1.89 2.45	7.91 14.2	6.08 19.1
M_R_ (emu/g)	700 1000	3.98 14.1	1.55 8.14	0.142 0.695	1.11 9.82	0.98 1.55
H_C_ (Oe)	700 1000	49.5 131	4.62 12.8	1.81 10.1	10.4 14.7	20.8 51.6
S	700 1000	0.437 0.475	0.192 0.727	0.075 0.284	0.143 0.692	0.161 0.081
K × 10^3^ (erg/cm^3^)	700 1000	0.286 2.44	0.023 0.090	0.002 0.015	0.052 0.131	0.079 0.619

## Data Availability

Not applicable.
